# Depression and postoperative complications: an overview

**DOI:** 10.1186/s12893-016-0120-y

**Published:** 2016-02-02

**Authors:** Mohamed M. Ghoneim, Michael W. O’Hara

**Affiliations:** Department of Anesthesia – 6JCP, University of Iowa Hospitals and Clinics, Iowa City, IA 52242 USA; Department of Psychological and Brain Sciences, University of Iowa, Iowa City, IA 52242 USA

**Keywords:** Surgery, Depression, Postoperative complications

## Abstract

**Background:**

The interaction of depression and anesthesia and surgery may result in significant increases in morbidity and mortality of patients. Major depressive disorder is a frequent complication of surgery, which may lead to further morbidity and mortality.

**Literature search:**

Several electronic data bases, including PubMed, were searched pairing “depression” with surgery, postoperative complications, postoperative cognitive impairment, cognition disorder, intensive care unit, mild cognitive impairment and Alzheimer’s disease.

**Review of the literature:**

The suppression of the immune system in depressive disorders may expose the patients to increased rates of postoperative infections and increased mortality from cancer. Depression is commonly associated with cognitive impairment, which may be exacerbated postoperatively. There is evidence that acute postoperative pain causes depression and depression lowers the threshold for pain. Depression is also a strong predictor and correlate of chronic post-surgical pain. Many studies have identified depression as an independent risk factor for development of postoperative delirium, which may be a cause for a long and incomplete recovery after surgery. Depression is also frequent in intensive care unit patients and is associated with a lower health-related quality of life and increased mortality. Depression and anxiety have been widely reported soon after coronary artery bypass surgery and remain evident one year after surgery. They may increase the likelihood for new coronary artery events, further hospitalizations and increased mortality. Morbidly obese patients who undergo bariatric surgery have an increased risk of depression. Postoperative depression may also be associated with less weight loss at one year and longer. The extent of preoperative depression in patients scheduled for lumbar discectomy is a predictor of functional outcome and patient’s dissatisfaction, especially after revision surgery. General postoperative mortality is increased.

**Conclusions:**

Depression is a frequent cause of morbidity in surgery patients suffering from a wide range of conditions. Depression may be identified through the use of Patient Health Questionnaire-9 or similar instruments. Counseling interventions may be useful in ameliorating depression, but should be subject to clinical trials.

## Background

Virtually no one escapes having a condition for which effective treatment requires anesthesia and surgery [1]. Around 15 % of patients who undergo surgery are at high risk of complications, leading to 80 % of all perioperative deaths. Those who survive suffer a substantial reduction in functional independence and long-term survival [2]. Therefore, precise evaluation of each patient’s risk is important to allow informed decision making by the patient prior to surgery and target specific interventions which may improve the outcome.

Major depressive disorder (MDD) or depression is a heterogeneous disease characterized by low mood, loss of interest and pleasure in normally enjoyable activities, loss of energy, difficulties in thinking and decision-making, appetite and sleep disturbances, psychomotor disturbances and suicidal ideation [3]. Anxiety is a frequently co-morbid [4]. In high income countries the average lifetime prevalence is approximately 15 % [5]. The World Health Organization (WHO) ranks MDD as the fourth leading cause of disability worldwide and projects that by 2030, it will be the second leading cause [6]. MDD represents a serious and often recurrent disorder. In addition to reduced general functioning and quality of life, depressed patients suffer from increased physical morbidity and mortality [7, 8]. The aetiology of the disease is not well understood, and the rates of its diagnostic recognition are poor (35–45 %) [9]. Furthermore, only a minority of diagnosed patients are provided with adequate treatment. Ormel, et al. [10] found that in high-income countries severely disabling mental disorders are only half as likely to be treated compared to serious disabling physical disorders.

## Literature search

Literature search was conducted in the following electronic databases: PubMed, CINAHL, Cochrane Library, PsycNet, Scopus, Web of Knowledge and NCBI. The following search terms were used: depression and surgery, depression and postoperative complications, depression and postoperative cognitive impairment, depression and cognition disorder, depression and intensive care unit, depression and mild cognitive impairment, and depression and Alzheimer’s disease. The search was started in June, 2012 down to 1982. We updated the search from June 2012 to April 2014 in Pub Med only. Articles in English language were the only ones pursued. Despite a sizable amount of literature that was examined, we did not run across a single comprehensive review of the interactions of depression with surgery as opposed to interactions with single types of surgeries, e.g., cardiac, bariatric, etc.

In this review we discuss the effects of depression on immunity, cognition and acute and chronic postoperative pain. We follow with discussion on the incidence of postoperative delirium and in patients being cared for in intensive care units. Then we discuss the interaction of depression with some specific surgeries. We conclude with recommendations for management of the depressed patient and future research.

## Review of the literature

### Immunity, depression and surgery

The immune system helps to protect the body against disease, toxic agents, stress and injury. Psychological stress in humans induces an inflammatory response through the production of small messenger molecules, pro-inflammatory cytokines such as interleukin 1beta (IL-1β), interleukin 6 (IL-6) and tumor necrosis factor alpha (TNFα). All of these are released by macrophages, other peripheral immune cells and microglia, as part of the early acute phase reaction to combat insult or threat [11, 12]. The state of immune depression is characterized by a depression in T-cell function and a shift toward a T_H_2 T-cell phenotype [13].

The immune mechanisms involved in the pathophysiological processes include networks between endocrine, autonomic (the hypothalamic-pituitary-adrenal axis) and neurotransmitter systems [14]. Bufalino, et al. [15], reviewed recently the existing literature on the genetic variants within key elements of the inflammatory system and the risk of depression. There was evidence for the involvement of genes related to inflammatory cytokines and enzymes (COX-2 and PLA2) in the aetiology of depression. In addition, single nucleotide polymorphisms (SNPs) in genes related to the serotonin pathway may play a fundamental role in the shared genetic liability to both depressive symptoms and immune activation. Decreased immunity may increase the risk of postoperative infections and cancer growth in patients harboring malignant tumors.

### Surgery and postoperative infections

Tissue injury following surgery results in depressed immunity leading to an increased risk of infectious complications. Postoperative infections are major causes of increased morbidity, mortality and cost [16]. Several studies reported increased rate of postoperative infections in patients suffering from depression, e.g., after coronary artery bypass surgery (CABG) [17], total knee arthroplasty [18], craniotomies [19], insertion of ventricular assist devices [20]. The latter is of particular serious consequences.

### Immunity and malignancy

Immunity also plays an important role in cancer development, growth and progression. In the setting of human cancer, myeloid-derived suppressor cells (MDSCs) increase in the body including the tumor site [21]. MDSCs and tumor cells form a feedback loop that promotes tumor growth and progression [22]. MDSC release factors that inhibit immunity by blocking T-cell and NK-cell (types of lymphocytes) effector functions, attenuate T-cell migration and enhance angiogenesis, ultimately, promoting tumor growth and progression [23, 24].

Although some human studies [25] suggest that patients with depression may be at a higher risk for developing cancer, a larger number of studies [26, 27] suggest that it is unlikely that depression is an independent risk factor for cancer. Some studies reported high prevalence of depression in patients with malignancies [28, 29], while others found the rates of major depression to be similar to those of patients in primary care [30]. This does not contradict the fact that depressive symptoms are commonly experienced by most people in response to sad or stressful life events such as their diagnoses with cancer and its often aggressive and debilitating treatments.

In addition to depression affecting the quality of life [31], the mortality of the cancer patient is increased [29, 32]. The causes may be biological, e.g., genetic predisposition, decreased immunity and/or psychosocial. Depressed patients are more likely to smoke [33, 34], or to be alcohol dependent [35] and to be non-compliant with treatments. Spiegel, et al. [36], studied the effect of psychosocial treatment on survival of patients with metastatic breast cancer. One group was randomized to receive group psychotherapy and the other served as a control. After 20 months, patients in the study group lived one and a half year more than those in the control group. La Raja, et al. [37], compared patients who had undergone surgery for breast carcinoma followed by psychotherapy and a control group. After 1 year 94 % of the patients were free of malignancy, compared to 80 % in the control group. More studies in this area are needed.

### Depression and postoperative cognitive impairment

Depressive symptoms flag an increased and robust likelihood of cognitive decline and conversely, cognition at baseline is related to the course of depressive symptoms. The cognitive impairment typically consists of episodic memory impairment, poor attention, decrement of visuospatial skills, information processing and executive dysfunction [38, 39]. As a result, depression may have a major adverse effect on every day functioning. Depression is also a strong risk factor for normal subjects progressing to mild cognitive impairment (MCI) [40]. The construct of MCI identifies the intermediate state of cognitive function between the changes seen in aging and those fulfilling the criteria for dementia of Alzheimer’s disease (AD) [41]. The occurrence of depressive symptoms also constitute a predictor for patients who are more likely to progress to Alzheimer’s dementia [42].

The syndrome, postoperative cognitive impairment (POCD), is a relatively serious complication of anesthesia and major surgery for elderly patients over 60 years of age. An early cognitive decline in the first week post-surgery of about 30–50 % of patients is followed by a persistent decline in 10–20 % at 3 months postoperatively. It leads to problems with learning, memory, attention and concentration which may affect performance of daily activities and may result in early retirement and resignation from work [43]. Factors related to vascular disease are important for both POCD and depression [43, 44].

As depression commonly leads to cognitive impairment, one may expect that depression should be accounted for in all studies of POCD, however, a recent review of the literature [43] shows otherwise. In the absence of a controlled and randomized study design, it is reasonable to recommend the measurement of depression in POCD trials.

### Pain and depression

Pain has been viewed as a multidimensional experience with sensory, motivational and affective components [45]. There is the experience of the sensory component which includes the perception of location, quality and intensity of the noxious stimulus and the emotional dimension which processes the affective salience or unpleasantness of the stimulus. When pain and depression occur together, they result in worsening of both conditions [46].

### Acute postoperative pain

Many patients suffer from moderate to severe postoperative pain despite recent improvements in pain treatment. Severe pain is associated with decreased patient satisfaction, delayed postoperative ambulation, the development of chronic postoperative pain, increased incidence of pulmonary and cardiac complications, and increased morbidity and mortality [47, 48]. Relatively few studies have investigated the relationship between acute postoperative pain and psychological morbidity. Taenzer, et al. [49] found that depression prior to surgery was significantly correlated with postoperative pain measurements and analgesic requirements. De Cosmo, et al. [50] found that patients with preoperative anxiety and depression had higher pain intensities postoperatively and larger consumption of tramadol.

It is possible also that adequate postoperative analgesia may play a part in protection against postoperative depression. Royse, et al. [51] investigated the effects of high thoracic epidural analgesia versus a patient controlled intravenous analgesia for 3 days in patients following coronary artery bypass surgery (CABG). High thoracic epidural analgesia, which provides excellent pain relief to the majority of patients, resulted in a lower risk of depression for 6 months or more following surgery compared to patients who received patient controlled intravenous analgesia which provides a much greater variability for pain relief [52].

### Chronic post-operative pain

Chronic postsurgical pain (CPSP) is a serious clinical problem [53]. It is a common reason for early retirement and unemployment and represents an extensive drain on societies' resources [54, 55].

The factors that seem to affect its incidence include the extent of preoperative pain, trauma during surgery and anxiety and depression [56, 57]. Cancer patients seem particularly susceptible [58]. Depression affects 30–100 % of patients with chronic pain [53]. Hinrichs-Rocker, et al. [59] systematically reviewed the psychosocial predictors and correlates for chronic post-surgical pain. Depression was a strong predictor. Adogwa, et al. [60] and Chaichana, et al. [61] came to the same conclusion. Comorbidity of pain and depression provokes worsening of both conditions [62]. Chronic pain is difficult to treat. It is important therefore to identify patients at high risk for its development and optimize their management.

### Mechanisms linking pain and depression

Pain has been shown to cause altered synaptic connectivity at the prefrontal cortex [63] and hippocampus [64], as well as altered dopamine signaling from the ventral tegmental area [65]. These changes have been known to trigger negative symptoms of depression and may form the link between pain and depression [66]. More recently, dysfunction in the serotonergic (5HT) system has been suggested to plan an important role in the pathophysiology of both conditions. Lebe, et al. [67], demonstrated that pain after lumbar disc surgery modulates the association between 5HT gene polymorphisms and depression [67].

### Postoperative delirium and depression

Postoperative delirium is a clinical syndrome characterized simply by an acute change in mental status with a fluctuating course, a prominent disturbance in attention, and either disordered thinking or altered level of consciousness. It occurs in 15–53 % of surgical patients over the age of 65 years [68] and is associated with significant morbidity and mortality [69]. Although the pathophysiology of the relationship between the two conditions remains to be clarified, many studies have identified depression as an independent risk factor for postoperative delirium [70, 71]. Patients with preoperative depressive symptoms are more likely to develop postoperative delirium of a longer duration [72] and incomplete recovery to independent functioning after surgery [73].

### Depression and the intensive care patient

The introduction of intensive care units to manage patients who are critically ill after surgery led to their increased survival. However, some of the survivors may suffer from cognitive disabilities and other psychiatric disorders. Rincon, et al. [74] found a 13.7 % incidence of depression on the first day of admission to the ICU. Liberzon, et al. [75] demonstrated a 32 % incidence of depression or PTSD in patients who were admitted to the unit after abdominal aortic surgery. Sukantrat, et al. [76] reported 35 % and 47 % incidence of depression at 3 and 9 months respectively after discharge from the unit.

Davydow, et al. [77] conducted a review of depression in general intensive care unit (ICU) survivors. From 14 studies, the median point prevalence of “clinically significant” depressive symptoms was 28 %. Early post-ICU depressive symptoms were a strong risk factor for subsequent depressive symptoms and were associated with lower quality of life. Future studies should investigate the factors linking individual patients, critical illness and post-ICU recovery with depression. Myhren, et al. [78] studied 194 patients after their discharge from ICU. Optimism was a strong predictor for less anxiety and depression symptoms; while pessimism had the opposite effect. Intervention by clinical psychologists when needed during the ICU stay promotes patients’ recoveries from depression and other psychiatric complications [79].

Depression is common in patients with diabetes and increases the rate of complications from this disease [80]. Diabetic patients with two or more complications, especially neuropathy or nephropathy are at high risk of depression [81]. This knowledge can help clinicians to identify patients at risk of depression and facilitate early treatment. Patients with diabetes and comorbid depression have a greater risk of ICU admission. Davydow, et al. [82] suggested that improving depression treatment in patients with diabetes could potentially prevent hospitalization for critical illnesses and lower healthcare costs. Depression and anxiety preceding myocardial infarction were associated with increased incidence of cardiac complications in coronary care units and ICUs, following infarction [83]. Identification and treatment of depression may prevent these complications. Cerebral stroke is another cause for admission to ICU. Post-stroke depression is a very frequent complication with an estimated prevalence of as high as 80 % and with increased risk of mortality [84].

### Influence of depression on surgical mortality

In addition to increased rate of mortality in surgical patients with depression who were also suffering from heart disease [85, 86], malignancy [26, 87] or other specific causes [18], Abrams, et al. [88] investigated surgical patients admitted to ICUs. Anxiety and depression increased the risk of death. Cuijpers, et al. [89] also reported an increased risk of all causes of mortality in a meta-analysis of community studies. Several causes for the increased mortality have been suggested. One is non-compliance of depressed patients with medical recommendations [90, 91]. It has also been suggested that patients with existing psychiatric morbidity may be more likely to undergo surgery by a lower-quality surgeon [92]. Another factor is the tendency of depressed patients to lead a less than healthy lifestyle, e.g. tobacco smoking and excessive alcohol consumption [93]. Depressed patients may seek advice at a later stage of illness, lack effective communications with physicians and the delays resulting from treatment of depression before surgery [88].

Depression has a strong association with suicide. The probability of attempted or successful suicide in depressive people is much higher than for the general population [94, 95]. Bariatric surgery patients in particular have a higher suicide rate than the general population. Tindle, et al. [96] reported a completed suicide rate of 13.7 per 10,000 for male patients and 5.2 per 10,000 for female patients, compared to 2.4 and 0.7 respectively in the general U.S. population. Peterhänsel, et al. [94] reviewed the results of thirty studies concerning bariatric surgery and completed suicide. They estimated a suicide rate of 4.1/10,000 person-years, higher than the general population. As will be reported later when discussing bariatric surgery, obesity has a strong association with depression. Although for many patients their depression symptoms improve following surgery, they remain an issue for some patients.

### Depression and some specific surgeries

Patients who undergo different surgical procedures probably have different pathophysiologies which may affect their postoperative course. We chose three surgeries that have been adequately studied in their relation to depression namely, CABG, bariatric surgery and spine surgery.

### Depression and CABG

For some patients with coronary artery disease, despite successful surgery, the outcome can be disappointing, because of psychological impairments. The prevalence of depression approximates between 30 % and 40 % and it increases the risk of morbidity and mortality. It increases the incidence of postoperative delirium, unplanned hospital admissions and cardiac events, e.g., arrhythmias, return of angina symptoms [97, 98]. Doering, et al. [17, 73] studied patients at discharge from the hospital and 6 weeks later. Depressive symptoms were associated with infections, impaired wound healing, poor emotional and physical recovery, and impaired quality of life.

One consistent feature of the papers reviewed was the recommendation that clinicians ought to routinely assess patients’ depressive and anxiety symptoms prior to surgery which also conforms with the recommendation by the American Heart Association [99]. Future randomized and controlled studies are needed to confirm the value of these recommendations.

### Bariatric surgery and depression

Extreme obesity, characterized by a body mass index (BMI) of 40 kg/m^2^ or greater, is associated with significantly increased mortality, principally from cardiovascular disease, type 2 diabetes and several cancers [100]. It is also associated with increased risk of depression [101]. Surgically induced weight loss is a valid treatment.

### Depression in bariatric surgery candidates

Methodologic limitations, including the absence of well-defined control groups, the absence of randomization and the use of suboptimal psychometric measures [102], prevent definitive interpretation of the rates of psychopathology observed in bariatric surgery candidates. Questionnaires which assess self-report symptoms may be often biased by confounding covariates as compared to symptoms confirmed by an interview [103]. An epidemiological study of nearly 40,000 individuals found that persons who had a BMI of 40 kg/m^2^ or higher were nearly five times more likely to have experienced an episode of major depression in the past year than were individuals of average weight [104]. This and other findings strongly suggest that extremely obese individuals are more vulnerable to depression, although the factors responsible for this susceptibility are not clear. Weight related stigmatization and the emotional distress associated with the medical complications of morbid obesity may be contributors [105, 106]. Therefore, candidates for bariatric surgery should be assessed for depression and those in need should be treated.

### Changes after bariatric surgery

Most studies reported a significant decrease in depression and anxiety rating scores and improvement in quality of life scores after surgery. And there is usually a positive association between the decrease in the depression and anxiety scores and the amount of weight loss [107]. Deactivation of inflammatory pathways, normalization of HPA axis functioning, and reduction of psychological distress due to weight loss might be the explanation. Postoperative depression was associated with less weight loss at the 24–36 month follow-up assessment [103].

Some patients may regain some of the weight they had lost, usually within 2–4 years. The follow-up data of the Swedish Obese Subjects study [107, 108] showed that the depression scores increased again over time after a strong initial improvement. Importantly, greater weight loss was significantly associated with greater reduction of depressive symptoms in the long term. Close surveillance of patients might help to identify those who would benefit from interactions that target depression as a potential mediator for poorer weight loss outcome. It may also identify patients who harbor suicidal ideation.

### Depression and spine surgery

Lumbar discectomy is the most common surgical procedure for patients experiencing back and leg pain from herniated lumbar discs. However, not all patients will benefit; some will be vulnerable to poor clinical outcome [61], especially with revision surgery [60]. Depression and anxiety are common in many disease states of chronic pain, as was mentioned before and are potential predictors of surgical outcomes. Some studies reported high rates of preoperative depression [109, 110], and failed back surgery syndrome is a common problem with enormous costs to patients, insurers and society [111]. Its commonest cause is depression [60], which should be assessed and treated preoperatively to avoid another failed operation.

Löbner, et al. [109] in a longitudinal observation study, identified several factors which increase the risk of depression, e.g., females, older age, lower education level, etc. Future studies should include a non-surgery control group of patients together with the surgical cohort in a randomized fashion to build a consensus for screening and management of patients for disc surgery.

**Fig. 1:**
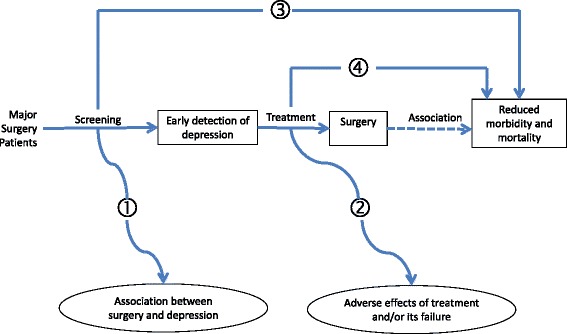
A framework for counselling interventions in major surgery patients. The numbers refer to answers to key questions as follows: (1) There are not enough studies comparing surgery with non-surgery cohorts. (2) There are potential adverse effects of treatment and/or its failure (3) and (4). There is a need for randomized and controlled trials to prove the efficacy of screening and/or treatment of depression before anesthesia and surgery in reducing postoperative morbidity and mortality.

A framework for counselling interventions in major surgery patients is detailed in Fig. 1.

### Clinical management of depression

Even in a busy surgical practice, it is relatively easy to screen for depression. There are numerous tools available that can be administered pre-operatively, e.g., the Patient Health Questionnaire-9 [112] which has 9 items that assess the symptoms of major depression,^3^ each of which are rated on a 4-point (0–3) scale. A score of 10 or above indicates that the patient is at high risk for major depression (sensitivity = .88; specificity = .88). For patients who screen positive, the next step is to do a more formal evaluation of the patient’s depression and to develop a treatment plan if indicated by a psychiatrist or clinical health psychologist. These types of consultations usually can occur quickly and should not ordinarily delay surgery. One major question will be whether the depression symptoms are largely reflective of the underlying disease for which the surgery is being performed, e.g., hypothyroidism, hyperparathyroidism or whether the symptoms reflect comorbid depression. For the surgeon (and patient) the question is whether to delay surgery until the patient has at least showed a response to the treatment. In cases which cannot be delayed, pharmacotherapy or cognitive behavior therapy can begin as soon as the patient is sufficiently recovered. For purely elective surgeries, the operation may be delayed until the patient has responded to treatment.

Unfortunately, it is not always the case that patients will respond to psychotherapy or medications quickly. Moreover, some patients will come to surgery with a long history of failed treatments for depression and will hold out little likelihood that a new round of depression treatment will be successful. To complicate matters more, perioperative use of serotonin reuptake inhibitors which are used for treatment may contribute to adverse postoperative outcomes which include death [113]. Following surgery it is recommend that patients should be screened for depression, particularly those who were identified as depressed before surgery. Again, referrals to consultation liaison psychiatry and clinical psychology can be made in the context of positive screens.

## Conclusions

The available literature suggests that depression is prevalent in patients before major surgery. Non-alleviated, it may predict increased morbidity and mortality after the operation. It may be associated with greater postoperative pain, higher incidence of postoperative infections, progression of malignant tumors, poor health-related quality of life as well as other complications. Accurate prediction of perioperative risk enables informed consent for patients before surgery, guides clinical decision making in the perioperative period and allows clinical audit [114]. Multiple tools are available [115], but unfortunately, none of them include major depression among the patient-related factors.

The evidence base for the detrimental effects of depression in the surgical patient consists mainly of relatively small studies which make chance findings more likely and prevent considering all possible contributing factors (Fig. 1). They are mostly single center studies and are therefore subject to confounding, particularly when they are non-blinded which is usually the case. It is not sufficient simply to demonstrate that poor clinical outcomes are more frequently associated with depression. Higher rates of psychiatric disorders in surgical than in non-surgical patients would suggest that anesthesia and surgery contribute to the emergence of psychiatric morbidity. Unfortunately, such studies are uncommon. Large multi-center randomized controlled trials are needed to confirm that screening and treatment of depression improve the outcome of surgery (unless randomization is deemed ethically improper). Until such studies have been performed, we must recognize the limitations of our database and make best use of them to reduce the perioperative risk in the patients.

Ultimately the etiology and the molecular nature of the brain deficits in depression need to be elucidated and new approaches to reverse them have to be found. No new antidepressant has been developed in the last quarter century. In the meantime more work is needed in the public health arena. A multimodal approach to perioperative care, and the use of clinical registries may help to improve the management of the surgical patient [116]. They provide a nationwide, prospective, observational database; therefore avoiding performance bias, which can occur if patients are recruited from selected surgeons and centers (Table 1). In the future, these efforts may change the potentially high-risk depressed surgical patient to a low or no risk one.

**Table 1:** Recommendations for enhancement of the recovery of the depressed surgical Patient

• Adoption of reliable and feasible screening process.
• Use of large multi-center randomized controlled trials to confirm the value of screening and treatment of depression.
• Adoption of a multi-modal approach of peri-operative care.
• Inclusion of major depression in risk stratification tools for predicting morbidity and mortality in adult patients undergoing major surgery.
• More use of clinical registries in studies for the depressed surgical patient.

## Abbreviations

AD: Alzheimer’s disease
BMI: body mass index
CABG: coronary artery bypass surgery
CPSP: chronic postsurgical pain
ICU: intensive care unit
MCI: mild cognitive impairment
MDD: major depressive disorder
MDSC: myeloid-derived suppressor cells
POCD: postoperative cognitive impairment
SNP: single nucleotide polymorphism
WHO: World Health Organization
